# Chemically Synthesized *Alcaligenes* Lipid A Shows a Potent and Safe Nasal Vaccine Adjuvant Activity for the Induction of *Streptococcus* *pneumoniae*-Specific IgA and Th17 Mediated Protective Immunity

**DOI:** 10.3390/microorganisms8081102

**Published:** 2020-07-23

**Authors:** Ken Yoshii, Koji Hosomi, Atsushi Shimoyama, Yunru Wang, Haruki Yamaura, Takahiro Nagatake, Hidehiko Suzuki, Huangwenxian Lan, Hiroshi Kiyono, Koichi Fukase, Jun Kunisawa

**Affiliations:** 1 Laboratory of Vaccine Materials, Center for Vaccine and Adjuvant Research, and Laboratory of Gut Environmental System, National Institutes of Biomedical Innovation, Health and Nutrition (NIBIOHN), Osaka 567-0085, Japan; k-yoshii@nibiohn.go.jp (K.Y.); hosomi@nibiohn.go.jp (K.H.); wan@nibiohn.go.jp (Y.W.); nagatake@nibiohn.go.jp (T.N.); suika3011a@yahoo.co.jp (H.S.); h-lan@nibiohn.go.jp (H.L.); 2 Graduate School of Medicine, Osaka University, Osaka 565-0871, Japan; 3 Department of Chemistry, Graduate School of Science, Osaka University, Osaka 560-0043, Japan; ashimo@chem.sci.osaka-u.ac.jp (A.S.); yamaurah18@chem.sci.osaka-u.ac.jp (H.Y.); koichi@chem.sci.osaka-u.ac.jp (K.F.); 4 Graduate School of Pharmaceutical Sciences, Osaka University, Osaka 565-0871, Japan; 5 International Research and Development Center for Mucosal Vaccines, the Institute of Medical Science, the University of Tokyo (IMSUT), Tokyo 108-8639, Japan; kiyono@ims.u-tokyo.ac.jp; 6 IMSUT Distinguished Professor Unit, the Institute of Medical Science, the University of Tokyo, Tokyo 108-8639, Japan; 7 Graduate School of Medicine, Chiba University, Chiba 260-8670, Japan; 8 Department of Medicine, School of Medicine and Chiba University-University of California San Diego (CU-UCSD) Center for Mucosal Immunology, Allergy and Vaccine, University of California, Oakland, CA 92093-0063, USA; 9 Graduate School of Dentistry, Osaka University, Osaka 565-0871, Japan; 10 Department of Microbiology and Immunology, Graduate School of Medicine, Kobe University, Hyogo 650-0017, Japan; 11 Graduate School of Biomedical and Health Sciences, Hiroshima University, Hiroshima 739-0046, Japan; 12 Research Organization for Nano & Life Innovation, Waseda University, Tokyo 162-0041, Japan

**Keywords:** *Alcaligenes* lipid A, IgA antibody, neutrophil, pneumococcal surface protein A (PspA), *Streptococcus pneumoniae*, Th17 response

## Abstract

Effective and safe vaccine adjuvants are needed to appropriately augment mucosal vaccine effects. Our previous study demonstrated that lipopolysaccharide (LPS) from Peyer’s patch resident *Alcaligenes* stimulated dendritic cells to promote the production of mucosal immunity-enhancing cytokines (e.g., IL-6 and BAFF), thus enhancing antigen-specific immune responses (including IgA production and Th17 responses) without excessive inflammation. Here, we chemically synthesized *Alcaligenes* lipid A, the biologically active part of LPS, and examined its efficacy as a nasal vaccine adjuvant for the induction of protectively immunity against *Streptococcus pneumoniae* infection. Mice were nasally immunized with pneumococcal surface protein A (PspA) as a vaccine antigen for *S. pneumoniae*, together with *Alcaligenes* lipid A. *Alcaligenes* lipid A supported the generation of high levels of PspA-specific IgA and IgG responses through the augmentation of germinal center formation in the nasopharynx-associated lymphoid tissue and cervical lymph nodes (CLNs). Moreover, *Alcaligenes* lipid A promoted PspA-specific CD4^+^ Th17 responses in the CLNs and spleen. Furthermore, neutrophils were recruited to infection sites upon nasal infection and synchronized with the antigen-specific T and B cell responses, resulting in the protection against *S. pneumoniae* infection. Taken together, *Alcaligenes* lipid A could be applied to the prospective adjuvant to enhance nasal vaccine efficacy by means of augmenting both the innate and acquired arms of mucosal immunity against respiratory bacterial infection.

## 1. Introduction

Various pathogens cause infectious diseases by invading through the surface of the respiratory and gastrointestinal tracts [1]. It is therefore important to induce protective immunity at mucosal sites to prevent these diseases. In vaccine development, the injection-type vaccines can induce systemic immune responses but not mucosal immune responses, whereas mucosal vaccines (e.g., nasal and oral vaccines) can induce both systemic and mucosal immune responses [2]. Therefore, mucosal vaccines can be considered to protect from infectious diseases caused by mucosally invading pathogens.

In the respiratory immune system, nasopharynx-associated lymphoid tissue (NALT) has been suggested to be one of target tissues for the delivery of nasal vaccine, since it is equipped with all of the necessary immune cells for the induction of antigen-specific immune responses. In murine nose, NALT is found as on both sides of the nasopharyngeal duct, dorsal to the cartilaginous soft palate, and it is considered analogous to Waldeyer’s tonsillar ring in humans [3]. In the NALT, nasal vaccines are taken up by M cells and transported to dendritic cells (DCs) for antigen processing and presentation, which leads to the induction of antigen-specific T and B cells, together with the formation of germinal centers [1,3]. In the germinal centers of NALT, immunoglobulin (Ig) M^+^ naïve B cells preferentially undergo class switch to IgA and differentiate into IgA^+^ plasmablast cells. IgA^+^ plasmablast cells then migrate to the nasal passages, where they differentiate into plasma cells that produce IgA antibodies. IgA antibodies are secreted into the nasal cavity through nasal epithelial cells and bind to pathogens and pathogenic materials (e.g., toxins), and thus prevent the development of infectious diseases.

It is generally known that as like the injection-type vaccines, nasal vaccination also requires a safe and effective mucosal adjuvant for the induction of protective immunity. Recently, mucosal adjuvant candidates of microbial components recognized by toll-like receptors (TLRs) have been developed, and some of them are in clinical trials. For example, the bacterial flagellin, including *Vibrio vulnificus* FlaB, acts as a nasal vaccine adjuvant to enhance the effects of nasal vaccines using inactivated influenza virus via TLR5-mediated activation [4]. Additionally, CpG oligodeoxynucleotides (CpG-ODNs) are used as a nasal vaccine adjuvant to enhance Th1 responses via the recognition of TLR9 by CPG-ODN, resulting in the protection of influenza virus infection [5,6,7,8].

Our previous study identified *Alcaligenes* as symbiotic resident bacteria inside Peyer’s patches (PPs), a major gut-associated lymphoid tissue in the small intestine [9]. In the PPs, *Alcaligenes* stimulated DCs to enhance the production of IgA-enhancing cytokines including interleukin (IL)-6 and a proliferation-inducing ligand (APRIL) [9]. A subsequent study focused on the stimulatory effects of bacterial lipopolysaccharide (LPS) of *Alcaligenes*, a Gram-negative bacterial component that acts as a TLR4 ligand [10]. We demonstrated that *Alcaligenes*-derived LPS stimulated DCs to enhance the production of IgA-enhancing cytokines, and thus promoted the production of IgA antibodies [10]. In addition, we showed that mice subcutaneously immunized with ovalbumin (OVA), together with *Alcaligenes*-derived LPS exhibited higher levels of OVA-specific IgG antibodies and greater Th17 responses than did mice subcutaneously immunized with OVA alone [10]. Although *Escherichia coli*-derived LPS is known to induce excessive inflammation, *Alcaligenes*-derived LPS showed biologically negligible inflammatory responses [10]. These findings suggested that *Alcaligenes* LPS could be an effective and safe adjuvant.

LPS is a biologically active component of gram negative bacteria, whose structure consists of a hydrophilic region of oligosaccharide (an O-antigen) and a hydrophobic region of lipid A [11,12]. Since LPS is a large and complex molecule and its O-antigen region occasionally induces pathogenic inflammation, it has been difficult to use whole LPS as a vaccine adjuvant. In contrast, lipid A—an active component of LPS to be recognized by TLR4—is a simple molecule, and some of these molecules and their analogues derived from various bacteria have already been chemically synthesized and used as vaccine adjuvants [13,14].

*Streptococcus pneumoniae* is a major pathogen in the respiratory tract, which causes various pathologies, including meningitis, sepsis, and pneumonia [15]. Pneumococcal surface protein A (PspA) is one of the potent candidates as a pneumococcal vaccine antigens, because of cross-reactivity among different strains [15]. As it is known that Th17 response is essential to prevent pneumococcal infection [16] and *Alcaligenes* LPS could induce antigen-specific Th17 responses [10], nasal vaccination of PspA with *Alcaligenes* lipid A is expected to efficiently induce protective immunity against *S. pneumoniae* infection.

In this study, we obtained chemically synthesized *Alcaligenes* lipid A and examined its efficacy as a nasal vaccine adjuvant against *S. pneumoniae*. Our current study indicated that *Alcaligenes* lipid A could be an efficient adjuvant for the nasal vaccines against respiratory infection with *S. pneumoniae*.

## 2. Materials and Methods

### 2.1. Mice

Female Balb/c mice (age, 6 or 7 weeks) were purchased from CLEA Japan (Tokyo, Japan) and kept for at least 1 week before the experiments. All experiments were conducted in accordance with the guidelines of the Animal Care and Use Committee of the National Institutes of Biomedical Innovation, Health and Nutrition (NIBIOHN) and the Committee on the Ethics of Animal Experiments of NIBIOHN (approval no. DS25-2, DS25-3). In accordance with the guidelines, murine condition was checked at least once a day and the mice were euthanized if their body weight reduction exceeded 20%.

### 2.2. Preparation of PspA Protein

The PspA gene was amplified by polymerase chain reaction (PCR) and cloned into pET16b plasmid (Novagen, Darmstadt, Germany), as previously described, to yield pET16b-PspA plasmid [17]. To obtain PspA recombinant proteins, the plasmids were transformed into *E. coli* strain BL21 (DE3) (Novagen). Protein production was induced by adding isopropyl-β-D-thiogalactopyranoside (Nacalai Tesque, Kyoto, Japan). The culture pellets were sonicated for 1 min three times in buffer A (10 mM Tris-HCl [pH 8.0], 400 mM NaCl, 5 mM MgCl_2_, 0.1 mM PMSF, 1 mM 2-mercaptethanol, and 10% glycerol). After centrifugation of the mixture at 4 °C and 17,800× *g* for 15 min, the supernatants were filtered through a 0.45 µm Millex-HV filter unit (Merck Millipore, Burlington, MA, USA) and loaded into HiTrap HP columns (GE Healthcare, Pittsburgh, PA, USA). PspA was eluted with buffer A containing 100 to 500 mM imidazole. The eluted protein was loaded into a PD-10 column (GE Healthcare) for exchange with PBS (Nacalai Tesque). The concentration of purified protein was measured by using a BCA protein assay kit (Pierce Chemical, Rockford, IL, USA). The purity of the eluted protein was confirmed in a NuPAGE electrophoresis system (Life Technologies, Carlsbad, CA, USA) followed by staining with Coomassie brilliant blue (Nacalai Tesque).

### 2.3. Preparation of Alcaligenes Lipid A

*Alcaligenes* lipid A was chemically synthesized as previously described [18]. It was dissolved in dimethyl sulfoxide (Nacalai Tesque), filtered in a Millex-LG 0.2 µm filter unit (Merck Millipore), and then sonicated for 5 min. It was then stored at −30 °C until used.

### 2.4. Immunization

Mice were nasally immunized with vehicle (PBS), 5 µg of PspA alone, PspA together with 1 µg of cholera toxin (CT), or various amounts of *Alcaligenes* lipid A in 15 µL of PBS, without anesthesia, three times at one-week intervals. One week after the last immunization, serum, nasal wash fluid, and BALF were collected. To collect serum, blood was collected from immunized mice and left on ice for 30 min, and then centrifuged at 4 °C, 3000× *g*, 10 min. Nasal wash fluid was obtained by flushing 200 µL PBS of 4 °C in an anterior direction via a cut in the trachea. BALF was harvested by placing 1 mL of PBS of 4 °C in both of the lungs, and then flushing and absorbing it twice or three times in a posterior direction from a cut in the trachea. These samples were stored at −80 °C until used.

### 2.5. ELISA

PspA-specific antibody production was determined by ELISA. Immunoplates (96-well) (Thermo Fisher Scientific, Waltham, MA, USA) were coated with 5 µg/mL PspA in PBS at 4 °C overnight. The plates were then treated with 1% bovine serum albumin (BSA) (Nacalai Tesque) in PBS for 2 h at room temperature, to prevent non-specific binding. After washing of the plates with PBS containing 0.05% Tween 20, two-fold serial dilutions of serum, nasal wash fluid, or BALF were added to each well, and the plates were incubated for 2 h at room temperature. After washing of the plates with 0.05% Tween 20 in PBS, goat anti-mouse IgA, IgG, IgG1, IgG2b, or IgG3 conjugated with horseradish peroxidase (SouthernBiotech, Birmingham, AL, USA; 1:4000) in 1% BSA and 0.05% Tween 20 in PBS was added to the wells and the plates were incubated for 1 h at room temperature. PspA-specific antibodies were detected by adding 3, 3′, 5, 5′-tetramethylbenzidine peroxidase substrate (SouthernBiotech) to the wells and incubating for 2 min. Then, 0.5 M HCl was added to the wells and the absorbance at 450 nm (OD_450_) was measured by using an iMark microplate reader (Bio-Rad, Hercules, CA, USA).

### 2.6. Streptococcus pneumoniae Culture and Infection Model

*Streptococcus pneumoniae* Xen10, which possesses a stable copy of the modified *Photorhabdus luminescens lux* operon at a single integration site on the bacterial chromosome (parental strain, A66.1 serotype 3; Caliper Life Sciences, Hopkinton, MA, USA), was cultured in brain-heart infusion broth (Becton, Dickinson and Company, Franklin Lakes, NJ, USA) at 37 °C under a 5% CO_2_ atmosphere with no aeration. After overnight culture, *S. pneumoniae* was collected by centrifugation at 4 °C, 9100× *g*, 3 min and washed twice with sterile PBS. One week after the final immunization, mice were nasally challenged with 5.0 × 10^6^ CFU (40 µL per mouse) of *S. pneumoniae* under anesthesia. The survival and body weight of the infected mice were monitored for 14 days. After euthanizing infected mice, lungs and BALF were then collected from these mice and the collected lungs were homogenized for 1 min in 1 mL sterile PBS (Nacalai Tesque). The samples were plated on blood agar (BD Biosciences, San Jose, CA, USA) coating with kanamycin (100 µg/mL: 40 μL) (Nacalai Tesque). After sample culture overnight at 37 °C, the numbers of bacterial colonies on the blood agar plate were counted.

### 2.7. Ex vivo Imaging of S. pneumoniae Infection

Bioluminescence signals of *S. pneumoniae* in whole lungs were detected by using an in vivo imaging system (IVIS) charge-coupled device camera (Xenogen, Alameda, CA, USA). Total photon emission from the whole lungs of each mouse was measured by using the LivingImage software package (Xenogen). Exposure time was defined as 5 min to acquire all images. Measurement values are shown as numbers of photons s^−1^ cm^2^ sr^−1^.

### 2.8. Immunohistological Analysis

NALT, CLNs, and lungs were collected from mice, washed with PBS on ice, embedded in Tissue-Tek OCT compound (Sakura Finetek Japan, Tokyo, Japan) and cut into 6 µm sections by using a CM3050 S cryostat (Leica Biosystems, Wetzlar, Germany). Tissue sections of NALT and CLNs were fixed for 1 min at room temperature in prechilled 100% acetone (Nacalai Tesque). The sections of lung tissue were fixed for 30 min at 4 °C in prechilled 95% ethanol (Nacalai Tesque), before being fixed in prechilled 100% acetone. Tissue sections were washed twice in PBS (each wash for 5 min), and then blocked with 2% newborn calf serum (NCS) (Equitech Bio, Kerrville, TX, USA) in sterile PBS for 30 min at room temperature in an incubation chamber. Tissue sections were incubated with primary antibodies in sterile PBS containing 2% NCS overnight at 4 °C in the incubation chamber. The tissue sections were then washed in PBS containing 0.1% Tween 20 and PBS (each for 5 min), and then stained with secondary antibodies in sterile PBS containing 2% NCS for 30 min at room temperature in the incubation chamber. After being washed twice with PBS for 5 min each time, the tissue sections were stained with 1 µM 4′, 6-diamidino-2-phenylindole (DAPI) (AAT Bioquest, Sunnyvale, CA, USA) for 10 min at room temperature in the incubation chamber. Finally, the tissue sections were washed twice in PBS for 5 min each time, mounted in Fluoromount (Diagnostic BioSystems, Pleasanton, CA, USA) and examined under a fluorescence microscope (BZ-9000; Keyence, Osaka, Japan). The following monoclonal antibodies and reagents were used for immunohistological analysis: purified rat anti-mouse B220 (RA3-6B2; BioLegend, San Diego, CA, USA; 1:100), biotin–PNA (Vector Laboratories, Burlingame, CA, USA; 1:100), Alexa Fluor (AF) 488-goat anti-rat IgG (Thermo Fisher Scientific; 1:200), Cy3-Armenian hamster anti-goat IgG (Jackson ImmunoResearch Laboratories, West Grove, PA, USA; 1:200), and AF546-streptavidin (Thermo Fisher Scientific; 1:200). Cell nuclei were visualized by staining with DAPI as described above.

### 2.9. T-cell Assay

Cells were collected from CLNs and spleens by filtration with 100 µm cell strainers (Corning, New York, NY, USA). These cells were treated with 1 mL red blood cell lysis buffer (0.16 M NH_4_Cl and 0.17 M Tris) for 1 min at room temperature. CD4^+^ T cells were purified by using anti-mouse CD4 (L3T4) magnetic beads and a MACS System (Miltenyi Biotec, Bergisch Gladbach, Germany). The purified CD4^+^ T cells were resuspended at 2 × 10^6^ cells/mL in RPMI 1640 medium (Sigma-Aldrich, St. Louis, MO, USA), supplemented with 10% fetal bovine serum (FBS; Gibco, Thermo Fisher Scientific), 1% penicillin–streptomycin mixed solution (Nacalai Tesque), 1% 100 mM sodium pyruvate solution (Nacalai Tesque), and 0.1% 2-mercaptoethanol (Gibco, Thermo Fisher Scientific). Purified CD4^+^ T cells (2 × 10^5^ cells/well) were cultured with 30 Gy irradiated splenic cells as antigen-presenting cells (1 × 10^4^ cells/well) from unimmunized mice for 4 days at 37 °C in 5% CO_2_ in the presence of 1 µg/mL PspA. After the incubation, the culture supernatant was collected to detect cytokines, including IFN-γ, IL-4, and IL-17, by using a BD cytometric bead array kit (BD Biosciences), in accordance with the manufacturer’s instructions. Numbers of proliferating T cells were determined by using CyQUANT Cell Proliferation Assay Kits (Invitrogen, Thermo Fisher Scientific, Waltham, MA, USA), in accordance with the manufacturer’s instructions.

### 2.10. Cell Isolation and Flow Cytometric Analysis

Immunized and challenged mice were killed under anesthesia conditions, to collect the nasal cavity by excision with scissors. The cells of the nasal mucosa were then collected by using sharp curettes in a dish filled with RPMI-1640 medium containing 2% NCS (Equitech Bio). These cell suspensions were filtered through 100 µm cell strainers (Corning). After treating with red blood cell lysis buffer for 1 min, cell samples were first incubated (15 min at room temperature) with anti-mouse CD16/32 monoclonal antibody (BioLegend; 93; 1:100) to avoid non-specific staining and with 7-aminoctinomycin D (7-AAD; BioLegend; 1:100) to detect dead cells and exclude them from the analysis. Then, the cell samples were stained with the following fluorescently labeled monoclonal antibodies for 30 min at 4 °C: fluorescein isothiocyanate – rat anti-mouse Ly6G (BioLegend; 1A8 1:100), APC rat anti-mouse CD45 (BD Biosciences A20; 1:100), APC-Cy7-rat anti-mouse CD11b (BioLegend M1/70; 1:100). Samples fluorescently labeled with monoclonal antibodies were fixed at 4 °C overnight with sterile PBS (Nacalai Tesque) containing 1% paraformaldehyde (Nacalai Tesque). Samples were examined by using a MACSQuant flow cytometer (Miltenyi Biotec, Auburn, CA, USA), and data analysis was performed with FlowJo 9.9 software (Tree Star, Ashland, OR, USA).

### 2.11. Statistical Analysis

Data are presented as means ± SD. Statistical analyses were performed by using one-way ANOVA followed by Tukey’s multiple comparison test (GraphPad Software, La Jolla, CA, USA). Statistical significance was established at *p* < 0.05.

## 3. Results

### 3.1. Alcaligenes Lipid A Enhances Nasally-Induced PspA-Specific Mucosal Immune Responses through the Formation of Germinal Centers in the NALT

**Figure 1:**
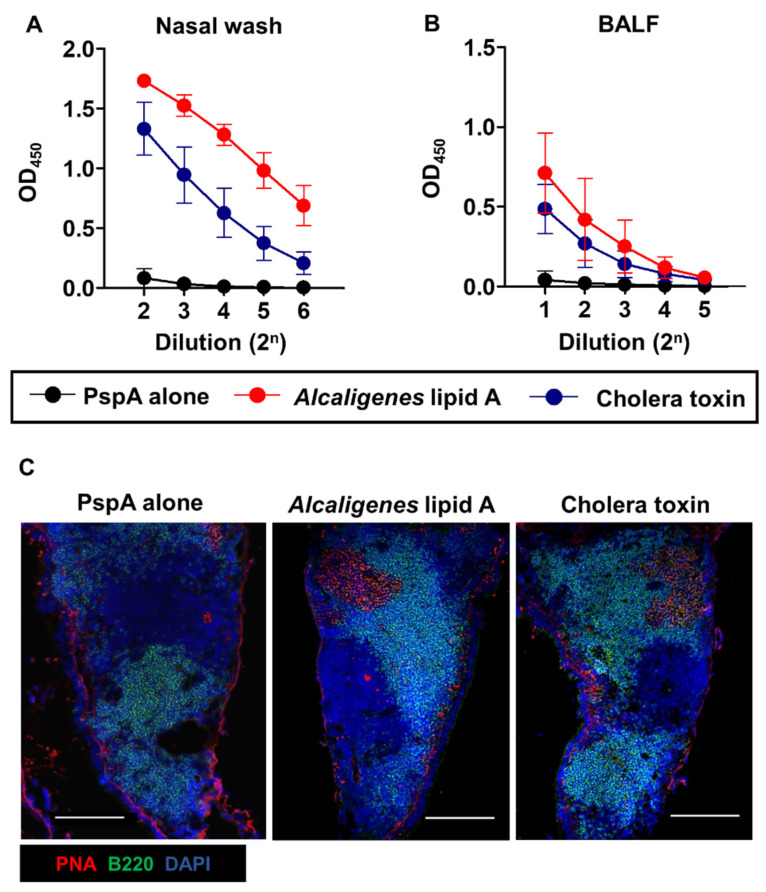
Nasal immunization with pneumococcal surface protein A (PspA), together with *Alcaligenes* lipid A enhances PspA-specific nasal immune responses through the induction of germinal center formation in the nasopharynx-associated lymphoid tissue (NALT). Mice were nasally immunized with PspA with or without *Alcaligenes* lipid A or cholera toxin three times at one-week intervals. One week after the final immunization, nasal wash (**A**) and bronchoalveolar lavage fluid (BALF) (**B**) were collected from immunized mice to measure the levels of PspA-specific IgA by enzyme-linked immunosorbent assay (ELISA). Data are representative of two independent experiments and are expressed as means ± SD (*n* = 6 per group). OD, optical density. (**C**) NALT was prepared, stained with the indicated antibodies and reagents, and then observed by using fluorescence microscopy. Images are representative of two independent experiments. Scale bars: 200 µm.

We first examined whether *Alcaligenes* lipid A promoted antigen-specific immune responses in the respiratory tracts of female Balb/c mice aged about 8 weeks. After nasal immunization with PspA together with *Alcaligenes* lipid A, nasal wash and bronchoalveolar lavage fluid (BALF) were collected to evaluate PspA-specific antibodies by enzyme-linked immunosorbent assay (ELISA). Mice nasally-immunized with PspA and *Alcaligenes* lipid A showed higher levels of PspA-specific IgA antibodies in the nasal wash and BALF than mice immunized with PspA alone (Figure 1A,B), demonstrating that co-administration of *Alcaligenes* lipid A supported the generation of elevated antigen specific-immune responses both in upper and lower respiratory tracts. The IgA enhancing adjuvant effects of *Alcaligenes* lipid A were a dose-dependent manner (Figure S1). Notably, *Alcaligenes* lipid A showed stronger IgA enhancing adjuvant activity than the classically used mucosal adjuvant cholera toxin (CT) [19].

Nasally co-administered *Alcaligenes* lipid A supported the induction of elevated PspA-specific antibody responses in upper respiratory tracts, so we next examined whether nasally-co-administered *Alcaligenes* lipid A contributed for the germinal center (GC) formation in the NALT, an important immunological event for initiation of antigen-specific immune responses in the nasopharynx cavity [20]. Histological analysis indicated that nasal immunization with PspA and *Alcaligenes* lipid A resulted in the GC formation with peanut agglutinin (PNA)^+^ B cells; this finding was similar to the NALT of mice nasally-immunized with PspA and classically known experimental mucosal adjuvant CT (Figure 1C). It should be noted that the GC formation was not observed in the NALT of mice nasally immunized with PspA alone. These findings demonstrated that *Alcaligenes* lipid A possessed a potent immune-enhancing activity by inducing GC formation in the key inductive site, NALT for the generation of antigen-specific IgA B cells.

Given the enhancement of PspA-specific IgA antibody production in lower respiratory tract (i.e., lung), we then examined whether *Alcaligenes* lipid A could induce the formation of inducible bronchus-associated lymphoid tissue (iBALT), an inductive site of antigen-specific immune responses in the lung [21,22]. No iBALT formation was noted in mice receiving nasal immunization with PspA together with either *Alcaligenes* lipid A or CT (Figure S2), indicating that the enhancement of antigen-specific immune responses in the lower respiratory tract was independent of iBALT formation.

These findings indicated that *Alcaligenes* lipid A acted as an efficient nasal vaccine adjuvant to enhance antigen-specific mucosal immune responses in the respiratory tracts through the induction of germinal center formation in the NALT, but not the iBALT formation in the lung.

### 3.2. Nasally-Co-Administered Alcaligenes Lipid A Enhances Systemic PspA-Specific Antibody Productions

**Figure 2:**
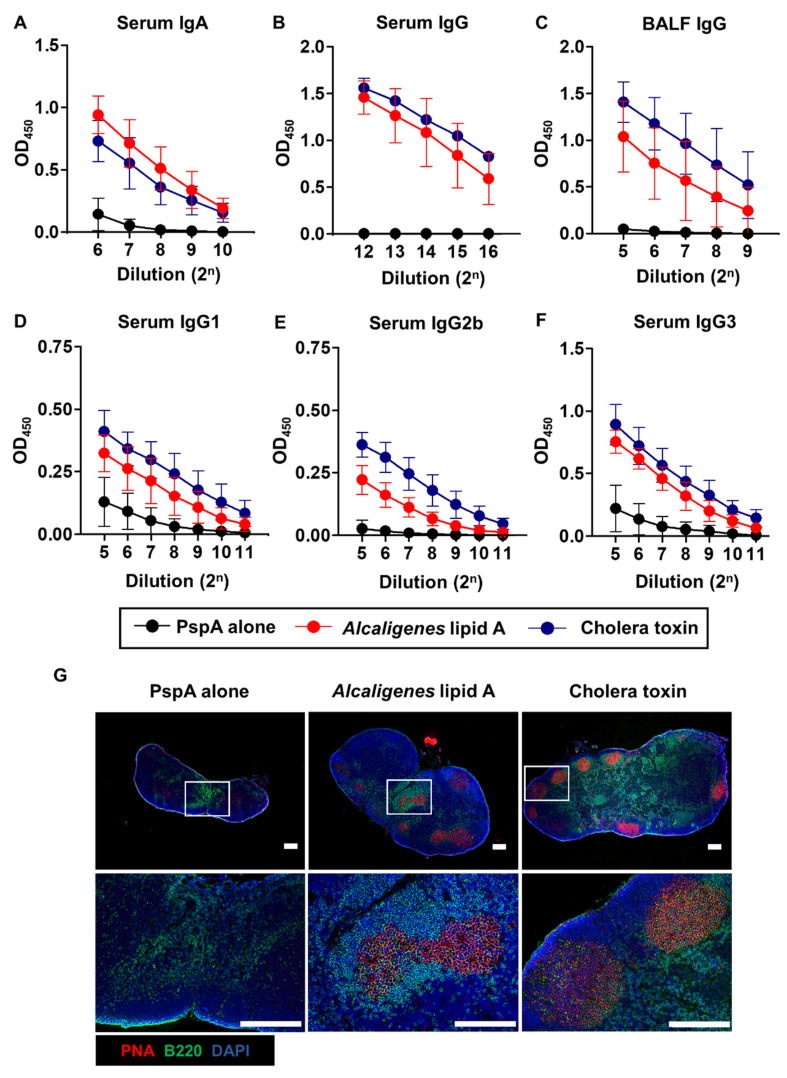
Nasal immunization with PspA, together with *Alcaligenes* lipid A enhances PspA-specific systemic immune responses. Mice were nasally immunized with PspA with or without *Alcaligenes* lipid A or cholera toxin three times at one-week intervals. One week after the final immunization, serum and BALF were collected from immunized mice to measure the levels of IgA (**A**) and IgG (**B**) in serum, IgG in BALF (**C**), and IgG subclasses in serum (**D–F**) by using ELISA. Data are representative of two independent experiments and are expressed as means ± SD (*n* = 6 per group). OD, optical density. (**G**) Cervical lymph nodes were prepared, stained with the indicated antibodies and reagents, and then observed by using fluorescence microscopy. Bottom panels are magnifications of the rectangles in the corresponding top panels. Scale bars: 200 µm.

We further examined whether nasal co-delivered *Alcaligenes* lipid A would enhance systemic antibody production. After nasal immunization of the mice with PspA and *Alcaligenes* lipid A, serum samples were collected for assessment of PspA-specific IgG and IgA antibodies by ELISA. Addition of *Alcaligenes* lipid A showed higher levels of PspA-specific IgG and IgA in serum from mice nasally immunized with PspA and *Alcaligenes* lipid A than mice received nasal PspA alone (Figure 2A). Subsequent analysis of PspA-specific serum IgG antibody subclass distributions reveled that *Alcaligenes* lipid A induced higher levels of PspA-specific IgG1, IgG2b, and IgG3 production than with PspA alone (Figure 2D–F). When the levels of PspA-specific IgG and IgA in the BALF were examined, higher levels of PspA-specific IgG and IgA were noted in mice nasally immunized with PspA and *Alcaligenes* lipid A than mice received nasal PspA alone (Figure 2B,C). In these systemic PspA-specific antibody responses, the adjuvant effects of *Alcaligenes* lipid A were also dose-dependent (Figure S3). 

Consistently, GC formation was detected in the cervical lymph nodes (CLNs)—draining lymph nodes associated with nasopharynx cavity, which serves as the interface with systemic immunity—of mice nasally immunized with PspA and *Alcaligenes* lipid A or CT (Figure 2G). In contrast, the GC formation was not observed in the CLNs of mice immunized with PspA alone (Figure 2G).

These results revealed that nasal co-administration of *Alcaligenes* lipid A activated systemic and respiratory PspA-specific antibody responses through the induction of GC formation in the draining lymph nodes (e.g., CLN).

### 3.3. Alcaligenes Lipid A Supported the Nasally-Induced PspA-Specific Th17 Responses in Both Mucosal and Systemic Compartments

**Figure 3:**
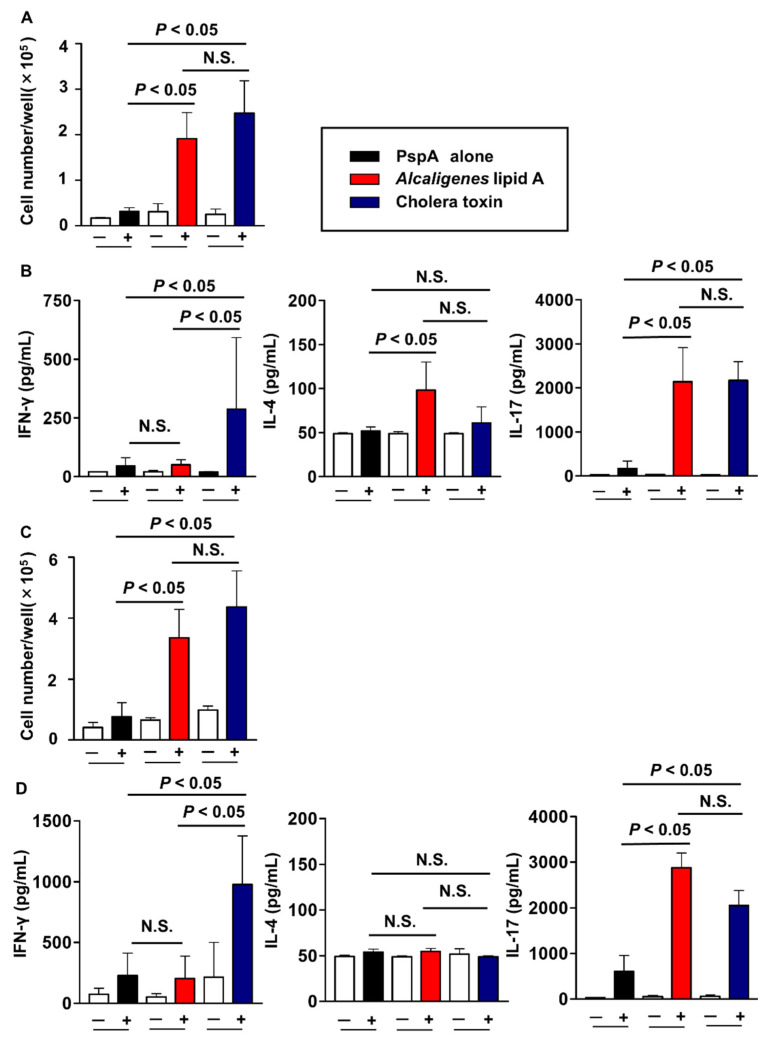
Preferential induction of Th17 responses in both respiratory and systemic compartments by nasal immunization with PspA together with *Alcaligenes* lipid A. Nasal immunization with PspA with or without *Alcaligenes* lipid A or cholera toxin, three times at one-week intervals. One week after the final immunization, CD4^+^ T cells were isolated from the cervical lymph nodes (CLNs) and spleen, and then cultured with irradiated antigen-presenting cells in the presence (+) or absence (−), respectively, of PspA. After 4 days, cultured cells were stained by using CyQUANT cell proliferation assay kits to calculate the numbers of proliferating T cells (**A**, CLNs; **C**, spleen). Data were combined from two independent experiments and expressed as means ± SD (*n* = 4 per group). The concentrations of IFN-γ, IL-4, and IL-17 in the culture supernatants were measured (**B**, CLNs; **D**, spleen). Data are representative of two independent experiments and are expressed as means ± SD (*n* = 4 per group). (+) and (−) indicate the presence or absence, respectively, of PspA during culture. Statistical significance was evaluated by using one-way ANOVA with *p* < 0.05; N.S., not statistically significant.

In as much as antigen-specific Th17 cells have been shown to play critical role for the protective immunity against *S. pneumoniae* infection^16^, we next investigated whether *Alcaligenes* lipid A could potentiate PspA-specific T cell responses. After nasal immunization of the mice with PspA and *Alcaligenes* lipid A, CD4^+^ T cells were isolated from the spleen and CLNs, cultured with PspA in the presence of antigen-presenting cells, and analyzed their proliferation and cytokine production. High levels of proliferation of antigen specific CD4^+^ T cells were noted in the CLNs from mice nasally immunized with PspA and *Alcaligenes* lipid A (Figure 3A). Similar results were obtained in mice nasally immunized with PspA and a classical standard mucosal adjuvant CT, whereas nasal immunization with PspA alone induced low levels of antigen specific CD4^+^ T cell proliferation (Figure 3A).

**Figure 4:**
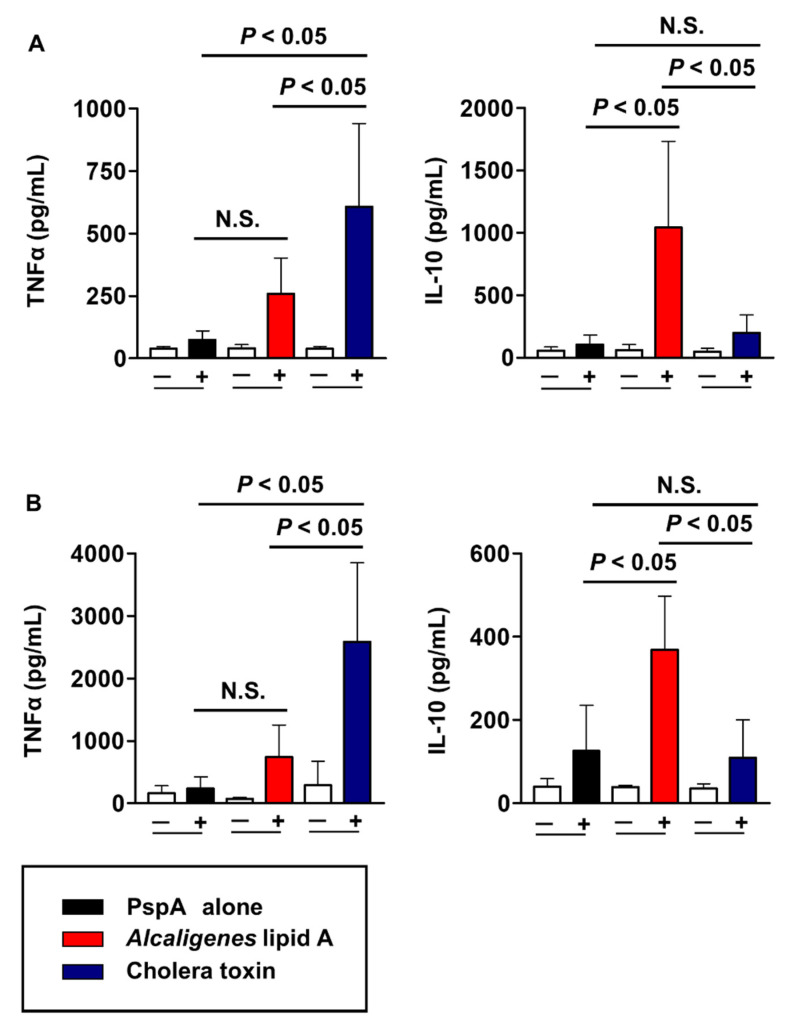
*Alcaligenes* lipid A suppresses a pro-inflammatory cytokine and enhances an anti-inflammatory cytokine. CD4^+^ T cells were isolated from the cervical lymph nodes **(A)** and spleen **(B)** of immunized mice and then cultured with irradiated antigen-presenting cells in the presence (+) or absence (−), respectively, of PspA. Culture supernatants were collected after 4 days to measure the concentrations of TNFα and IL-10. Data are representative of two independent experiments and are expressed as means ± SD (*n* = 4 per group). Statistical significance was evaluated by using one-way ANOVA at *p* < 0.05; N.S., not statistically significant.

Since *Alcaligenes* lipid A is a potent enhancer for PspA-specific CD4^+^ T cell responses, we next measured their cytokine production by CD4^+^ T cells. High levels of IL-17 production were detected in the CLNs of mice nasally immunized with PspA and *Alcaligenes* lipid A, whereas mice nasally immunized with PspA together with CT showed enhanced production of both interferon-gamma (IFN-γ) and IL-17 (Figure 3B). Similar results were obtained in the splenic CD4^+^ T cells from mice nasally immunized with PspA and either *Alcaligenes* lipid A or CT (Figure 3C,D). We further examined other cytokine production by CD4^+^ T cells in the CLNs and spleen of these nasally immunized mice. In both the CLNs and spleen, *Alcaligenes* lipid A induced significantly less production of pro-inflammatory TNFα but significantly greater production of anti-inflammatory IL-10, when compared with CT (Figure 4A,B). These findings indicated that *Alcaligenes* lipid A could preferentially induce PspA-specific CD4^+^ T cells producing either IL-17 or IL-10. 

### 3.4. Prevention of S. pneumoniae Infection by Nasal Immunization with PspA and Alcaligenes Lipid A

**Figure 5:**
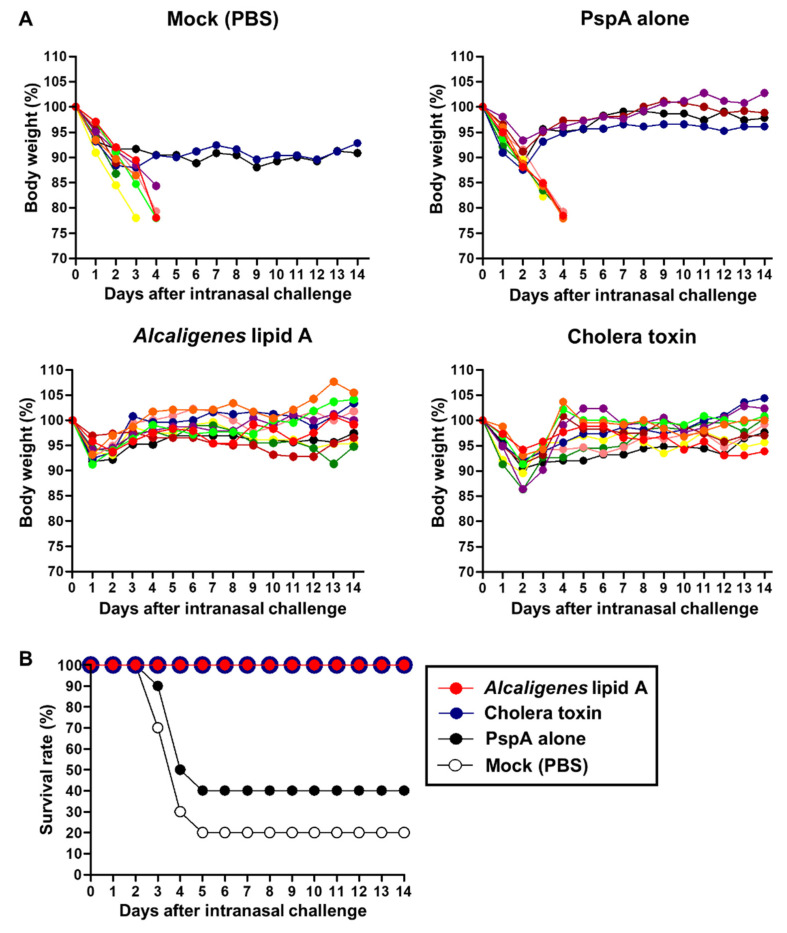
Nasal vaccination with PspA, together with *Alcaligenes* lipid A protects against *Streptococcus pneumoniae* infection. Nasal immunization with PspA with or without *Alcaligenes* lipid A or cholera toxin three times at one-week intervals. One week after the final vaccination, mice were nasally challenged with a lethal dose (5.0 × 10^6^ CFU/mouse) of *S. pneumoniae.* Their body weights (**A**) and survival rates (**B**) were then monitored for 14 days. The data are combined from two independent experiments (*n* = 10 per group).

Based on our findings on the adjuvant activity of *Alcaligenes* lipid A for the induction of PspA-specific mucosal and systemic immune responses (Figure 1 and Figure 2), we next investigated whether nasal immunization with PspA and *Alcaligenes* lipid A would induce protective immunity against *S. pneumoniae* infection. To test this, mice nasally immunized with PspA and *Alcaligenes* lipid A were nasally infected with *S. pneumoniae*. We then monitored these mice for 14 days to evaluate survival rates and changes in body weight. When mice had been nasally immunized with mock (i.e., phosphate buffered saline [PBS]) or with PspA alone, their body weights sharply decreased and many of them died (Figure 5A). Indeed, a few mice nasally immunized with mock (fewer than 20%) or PspA alone (fewer than 40%) survived after *S. pneumoniae* infection (Figure 5B). In contrast, when mice had been nasally immunized with PspA and either *Alcaligenes* lipid A or CT, their body weights declined during the first 2 days after the infection, but then sharply recovered to the normal body weight within 4 days, and all mice consequently survived (Figure 5A,B).

**Figure 6:**
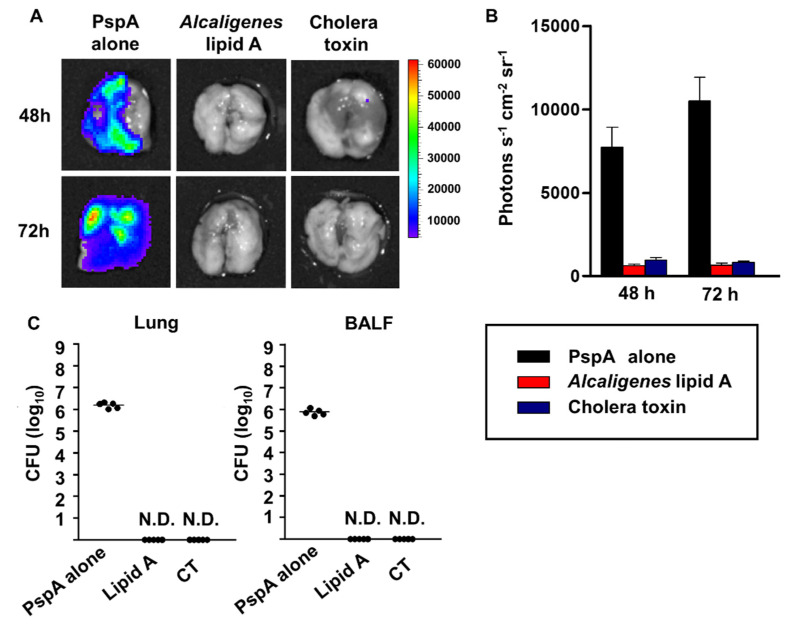
Prevention of bacterial growth in the lungs by nasal immunization with PspA plus *Alcaligenes* lipid A. 48 or 72 h after intranasal infection of mice with *S. pneumoniae*, lung tissue was collected for analysis of images (**A**) and average photon counts (**B**) of bioluminescence of *S. pneumoniae*. Similar results were obtained from two independent experiments (*n* = 5 per group). Data (**B**) are expressed as means ± SD. (**C**) Lung tissue and BALF were collected for enumeration of *S. pneumoniae* 72 h after infection. Data are representative of two independent experiments (*n* = 5 per group). The horizontal line indicates the median. CFU, colony-forming units; N.D., not detected.

The *S. pneumoniae* used in this study was genetically modified to express chemiluminescence [23], allowing us to check for the bacterium’s presence by *ex vivo* bioluminescence imaging [24]. The lungs of mice nasally immunized with PspA alone showed high intensity photon signals derived from *S. pneumoniae* (Figure 6A,B). In contrast, the lungs of mice nasally immunized with PspA plus either *Alcaligenes* lipid A or CT showed no bioluminescence signals (Figure 6A,B). Consistent with these findings, bacterial numbers in the lung and BALF from the mice vaccinated with PspA and either *Alcaligenes* lipid A or CT were significantly lower than those in mice vaccinated with PspA alone (Figure 6C).

**Figure 7:**
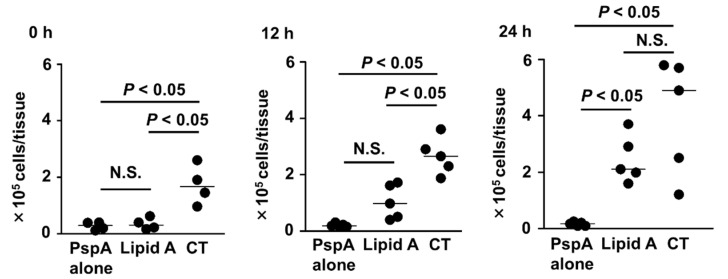
Infection-induced increase in neutrophil counts in the nose of mice nasally immunized with PspA plus *Alcaligenes* lipid A. Before or after *S. pneumoniae* infection (at 0 h, 12 and 24 h), cells were collected from the nasal mucosa for measurement of neutrophils. Data are representative of two independent experiments (uninfected, *n* = 4 per group; infected, *n* = 5 per group). The horizontal line indicates the median. Statistical significance was evaluated by using one-way ANOVA at *p* < 0.05; N.S., not statistically significant.

Th17 cells are known to recruit neutrophils for the protection from infection by extracellular bacteria, such as *S. pneumoniae* [25]. Thus, we investigated the neutrophil recruitments after infection. The numbers of neutrophils in the nasal mucosa increased in the 12 and 24 h after infection in mice nasally immunized with PspA and *Alcaligenes* lipid A (Figure 7). In contrast, small numbers of neutrophils were noted in any of the time periods in mice immunized with PspA alone (Figure 7). Notably, the neutrophil numbers in the uninfected condition (0 h) in mice nasally immunized with PspA and *Alcaligenes* lipid A was negligible (Figure 7). In contrast, the neutrophil numbers were elevated upon nasal immunization with PspA and CT before *S. pneumoniae* infection (Figure 7), suggesting that neutrophil recruitment into the nasal mucosa occurred regardless of bacterial infection, plausibly because of the inflammatory condition induced by immunization with CT, but not *Alcaligenes* lipid A. These findings collectively suggested that nasal vaccination with PspA and *Alcaligenes* lipid A induced neutrophil recruitment upon the *S. pneumoniae* infection, but not in the uninfected condition in the nasal mucosa.

## 4. Discussion

New mucosal adjuvants are needed to augment the immune responses when it is mucosally vaccinated with low-antigenic-subunit-ones. Moreover, it is rising the necessity of developing mucosal vaccines to protect various infectious diseases. We previously found that *Alcaligenes* LPS acts as a weak TLR4 agonist and enhances antigen-specific immune responses without excessive inflammation^10^. In this study, we extended these findings by showing that chemically synthesized *Alcaligenes* lipid A has strong potential as a safe and an effective nasal vaccine adjuvant to augment PspA-specific immune responses, and to prevent respiratory pneumococcal infection. These findings reveal that *Alcaligenes* lipid A acts as a vaccine adjuvant to enhance antigen-specific antibody production and Th17 responses without excessive inflammation, as well as *Alcaligenes* LPS.

Recently, a lipid A-based vaccine adjuvant has been developed. For example, 3-O-desacyl-4′-monophosphoryl lipid A (MPLA), a derivative of lipid A derived from *Salmonella minnesota* R595 LPS, is used for enhancement of the efficacy of Hepatitis B virus; human papillomavirus vaccine [26,27,28]. *S. minnesota* is a pathogenic bacteria, so its lipid A needs to be chemically modified to reduce its pathogenicity by deficient of phosphoryl group. In contrast, *Alcaligenes* is lymphoid resident commensal bacteria, thus, its lipid A could be applied to a vaccine adjuvant without chemical modification, and enhance antigen-specific immune responses without excessive inflammation.

It is known that the biological activity of lipid A differs among bacteria [12], and it is determined the differences in structure, such as in the length and linkage of fatty acids [29]. For example, *E. coli*-derived lipid A possesses six fatty acid chains and acts as a strong TLR4 agonist, consequently inducing excessive inflammation [30]. In contrast, *Bacteroides dorei* lipid A possesses four fatty acid chains and works as a TLR4 antagonist to competitively inhibit TLR4-mediated immuno-stimulatory activity by *E. coli* lipid A [31]. We have determined the structure of *Alcaligenes* lipid A that possesses the same number of acyl chain and phosphoryl group as *E. coli* lipid A [18], which possesses strong inflammatory activity. However, the length of acyl chain and the number of hydroxyl group was different between *E. coli* lipid A and *Alcaligenes* lipid A. Therefore, it is important to investigate the structure-activity relationship of *Alcaligenes* lipid A by using its derivatives to regulate immune responses by utilizing lipid A, which is a subject for future study.

In the present study, we demonstrated that nasal immunization with PspA and *Alcaligenes* lipid A enhanced PspA-specific IgA production in both the upper and lower respiratory tracts (Figure 1A,B). The enhancement of IgA production is associated with GC formation in the NALT [32]. GC formation needs an increase in the numbers of GC B cells in the NALT, and the main contributors to this increase are follicular helper T (T_FH_) cells [33,34]. When antigen-presenting cells activate T cells in the T cell zone of NALT, they are trafficked to the follicle outer edge of B cell follicle and differentiated into the T_FH_ cells [35]. T_FH_ cells express high levels of PD-1 and CXCR5, allowing their preferential accumulation from the follicle outer edge to the germinal center zone of B cell follicle by CXCL13 [36,37] derived from stromal cells and their interaction with B cells through CD40L-CD40 interaction [38,39,40]. Together with T_FH_-derived IL-21 stimulation, these reactions promote the GC formation, which leads to IgA class-switch recombination through the upregulation of activation-induced cytidine deaminase, the subsequent proliferation of IgA^+^ B cells, and their differentiation into IgA^+^ plasma cells [41,42]. Moreover, it has been demonstrated that Th17 cells play an important role in T-cell-dependent high-affinity IgA production in the PPs through the trans-differentiation into T_FH_ cells [43]. In the current study, we found that *Alcaligenes* lipid A induced Th17 cells (Figure 3B). This evidence and our results suggested that *Alcaligenes* lipid A can promote the differentiation of IgA^+^ B cells into IgA plasma cells via increases in Th17, T_FH_, and GC B cells in the NALT and CLNs.

We showed here that CT induced IFN-γ, IL-17, and TNFα production in CD4^+^ T cells in the CLNs and spleen (Figure 3B,D, and Figure 4). These subsets are known to be pathogenic Th17 cells involved in the onset and exacerbation of autoimmune diseases [44,45]. Unlike CT, *Alcaligenes* lipid A induced T cells producing IL-10 and IL-17 (Figure 3B,D, and Figure 4). IL-10 producing T cells suppress pro-inflammatory responses [46], and IL-10 also acts as IgA-enhancing cytokine [34], thus suggesting that *Alcaligenes* lipid A contributes to the promotion of IgA antibody production without excessive inflammation through the enhancement of IL-10 production. Recent evidence has indicated that IL-17- and IL-10-double positive Th cells existed in the small intestine; these are categorized as non-pathogenic (and possibly homeostatic) Th17 cells that suppress excessive inflammation [47]. Although it was known that non-pathogenic Th17 cells are differentiated by the stimulation with IL-6 and TGF-β derived from antigen presenting cells [48], the detail mechanism of their differentiation has not yet been fully elucidated. However, we have found that *Alcaligenes* lipid A stimulates bone marrow-derived DCs to promote IL-6 and IL-10 production in vitro (unpublished data), and this production is known to induce Th17 and IL-10-producing T cells [49,50]. Thus, these findings suggest that *Alcaligenes* lipid A is likely create an immunological environment that preferentially induces non-pathogenic Th17 differentiation in the nose.

Nasal immunization with PspA plus *Alcaligenes* lipid A induced IgG responses in addition to IgA (Figure 2). IgG antibodies bind to antigens, induce complement activation, opsonize extracellular bacteria, and promote phagocytosis through Fcγ receptors (FcγRs) expressed on neutrophils and macrophages [51,52]. Among the IgG subclasses, IgG3 antibodies are highly potent in the opsonophagocytosis, complement activation, and neutralization [53], and these were induced here by *Alcaligenes* lipid A in the case of *S. pneumoniae*. Moreover, it was reported that IgG antibodies bind to PspA to activate the C3 complement component, leading to the killing of *S. pneumoniae* [54]. Thus, it is likely that, in addition to the direct neutralization of *S. pneumoniae*, nasal immunization with PspA and *Alcaligenes* lipid A can generate antigen-specific IgG3-PspA complex on the bacteria to accelerate C3 complement and phagocytosis through FcγRs, resulting in the direct killing of *S. pneumoniae*.

In the current study, our results indicated that *Alcaligenes* lipid A induced neutrophil recruitments to infection sites (i.e., the nasal mucosa) (Figure 7). It was reported that IL-17 promoted the chemokine (e.g., CXCL1, 2, and 5) production from the epithelial cells mediated by IL-17R, thus promoting neutrophil recruitment to infection sites [55,56,57]. Moreover, a previous report has indicated that IgA triggers neutrophil migration to infection sites by interacting with IgA and FcαRI expressed on neutrophils, and this leads to additional neutrophil recruitment through the production of leukotriene B4 [58]. These findings collectively suggest that *Alcaligenes* lipid A promotes neutrophil recruitments to infection sites by both Th17 cells and IgA production.

Notably, low numbers of neutrophils were noted in mice receiving nasal immunization with PspA, together with *Alcaligenes* lipid A under uninfected conditions (Figure 7), suggesting that the recruitment of neutrophils seem to be mediated by antigen-specific immune responses, but not non-specific activation by lipid A, which was different phenotype from CT. We found that CT induced the accumulation of neutrophils in the nose under uninfected conditions, indicating that CT induced non-specific inflammation upon nasal administration. Consistent with this finding, it has been shown that CT induces inflammatory responses, including neutrophil infiltration into subepithelial tissues of nasopharynx cavity [59,60], and *E. coli* lipid A enhanced inflammatory cytokines, such as TNFα and IL-1β [61]. These findings suggest that *Alcaligenes* lipid A possesses higher levels of safety as a nasal vaccine adjuvant, compared with the classical mucosal adjuvant: CT and immuno-stimulatory component: *E. coli* lipid A.

There are some limitations to this study. The first limitation is the toxicity of *Alcaligenes* lipid A. Although we demonstrated that *Alcaligenes* lipid A did not induce excessive inflammation in this study, we have not yet fully investigated the increased dose, which indicates the toxicity of *Alcaligenes* lipid A. To utilize *Alcaligenes* lipid A safely, it is necessary to determine the dose showing toxicity, and set the optimal dose or chemically modify *Alcaligenes* lipid A structure to induce moderate immune response for clinical application in the future. The second limitation is to consider the influence of physical barriers (e.g., mucus and cilia) on their surface, which affects vaccine efficacy, as we previously reported [62,63]. The third one is the differences in anatomical characteristics of the inductive sites in the nose. We showed that *Alcaligenes* lipid A enhanced antigen-specific immune responses through the germinal center formation in the NALT of the murine model. Since humans do not have NALT, it is difficult to anticipate the induction pathway of *Alcaligenes* lipid A-enhanced immune responses as a nasal vaccine in humans.

The clinical application of nasal vaccine is an important issue for the prevention of respiratory infectious diseases. CT and *E. coli* heat labile toxin were utilized as experimentally efficient mucosal adjuvants [64,65]. When they were applied to clinical trial upon detoxicated modification, some recipients showed unacceptable neurological toxicity, including Bell’s palsy or facial paralysis [66]. In addition, several lipid A-based adjuvants (e.g., MPLA) have been developed by the modification of pathogen-derived lipid A and are already clinically used [67,68]. Unlike these lipid A, *Alcaligenes* lipid A is derived from commensal bacteria residing inside of PPs, where it provides appropriate stimulation to enhance immunity. Thus, *Alcaligenes* lipid A is likely to possess an intrinsically appropriate structure as a vaccine adjuvant. Since *Alcaligenes* resides inside the PPs from mouse, monkey, and humans [9], and *Alcaligenes* lipid A also stimulates human immune cells [69], it is potentially useful for a nasal vaccine adjuvant in humans.

In conclusion, our findings indicate that *Alcaligenes* lipid A acts as an efficient nasal vaccine adjuvant to augment the IgA, IgG, and Th17 responses in both the respiratory and systemic compartments. This augmentation is sufficient to prevent *S. pneumococcal* infection and recruit neutrophils. In addition, *Alcaligenes* lipid A suppresses the excessive inflammation caused by non-specific activation. These results suggest that *Alcaligenes* lipid A could be applicable as a nasal vaccine adjuvant against extracellular bacterial infection.

## Supplementary Materials

The following are available online at https://www.mdpi.com/2076-2607/8/8/1102/s1, Figure S1: Nasal immunization with PspA together with *Alcaligenes* lipid A enhances nasal immune responses in a dose-dependent manner, Figure S2: No induction of iBALT formation in mice nasally immunized with PspA together with *Alcaligenes* lipid A, Figure S3: Nasal immunization with PspA together with *Alcaligenes* lipid A enhances systemic antibody responses in a dose-dependent manner.
