# Supplementary material for: Chemically Synthesized Alcaligenes Lipid A Shows a Potent and Safe Nasal Vaccine Adjuvant Activity for the Induction of Streptococcus pneumoniae-Specific IgA and Th17 Mediated Protective Immunity
Source: Microorganisms. 2020 Jul 23;8(8):1102. doi: 10.3390/microorganisms8081102 (PMC7464877; doi:10.3390/microorganisms8081102)
Supplement: Supplementary file 1 [file microorganisms-08-01102-s001.zip › Supplementary Figure (Yoshii et al).pptx]

## Slide 1
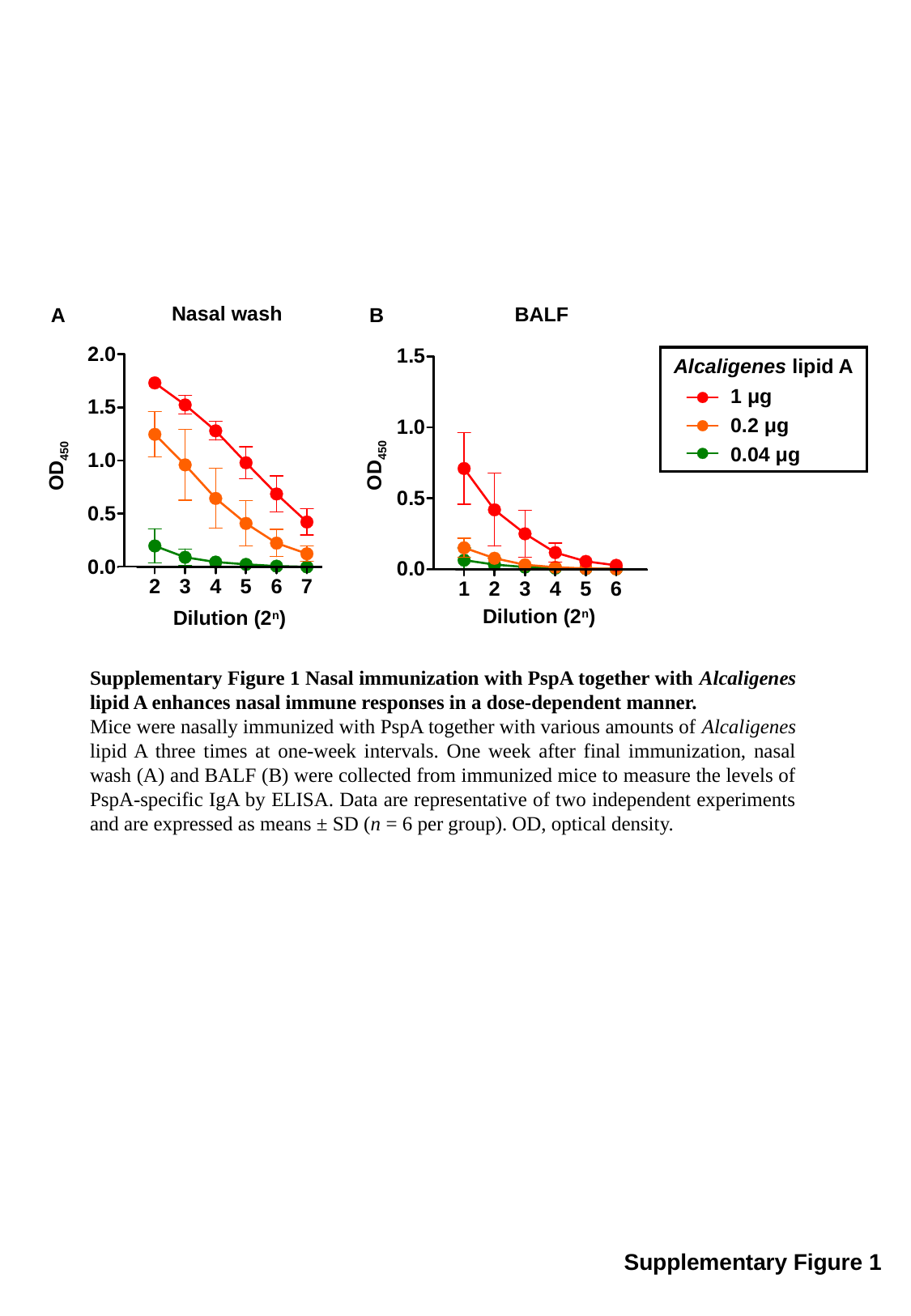

Nasal wash
BALF
A
B
OD450
OD450
Dilution (2n)
Dilution (2n)
Alcaligenes lipid A
1 μg
0.2 μg
0.04 μg
Supplementary Figure 1 Nasal immunization with PspA together with Alcaligenes lipid A enhances nasal immune responses in a dose-dependent manner.
Mice were nasally immunized with PspA together with various amounts of Alcaligenes lipid A three times at one-week intervals. One week after final immunization, nasal wash (A) and BALF (B) were collected from immunized mice to measure the levels of PspA-specific IgA by ELISA. Data are representative of two independent experiments and are expressed as means ± SD (n = 6 per group). OD, optical density.
Supplementary Figure 1

## Slide 2
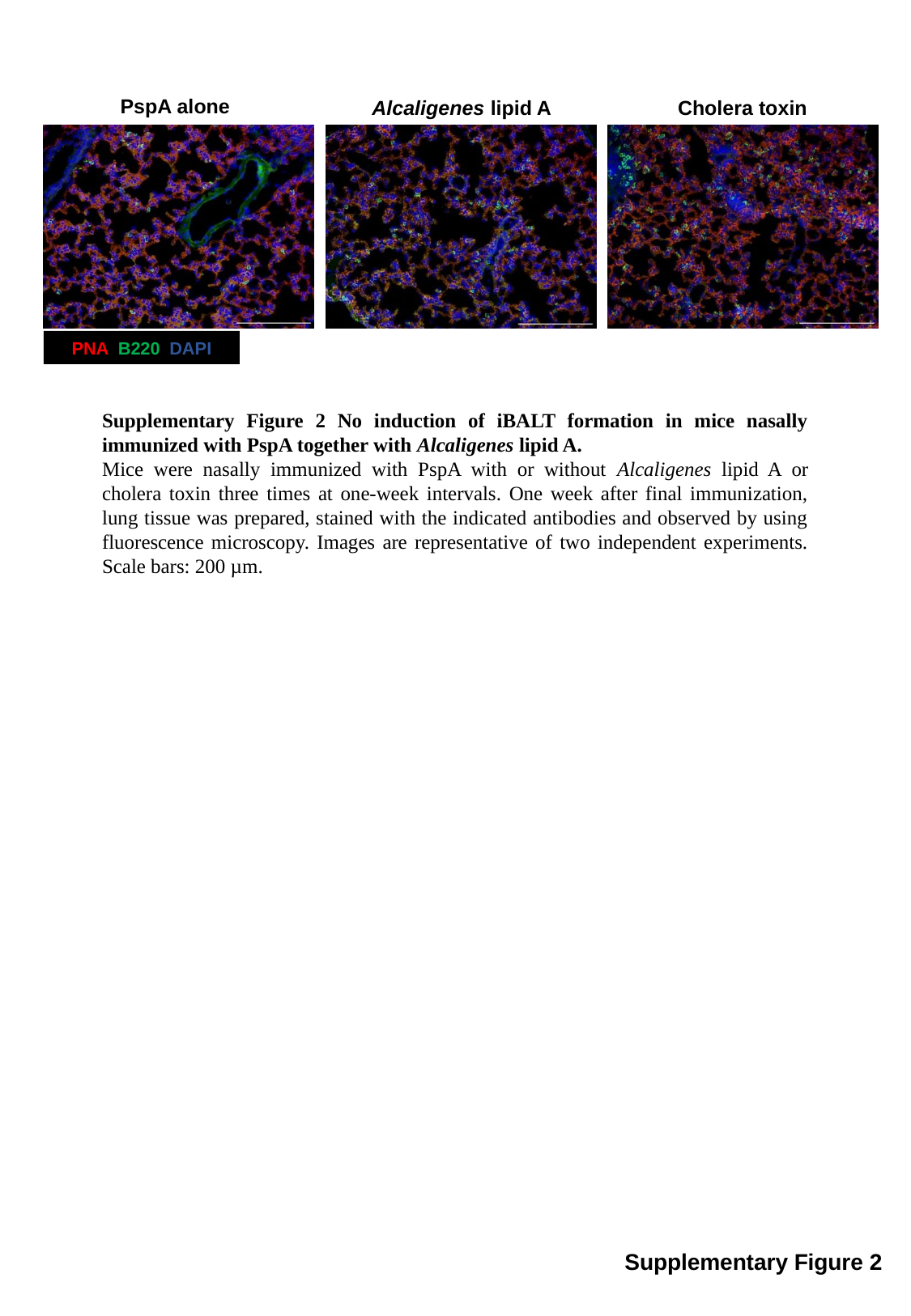

PspA alone
Cholera toxin
Alcaligenes lipid A
PNA B220 DAPI
Supplementary Figure 2 No induction of iBALT formation in mice nasally immunized with PspA together with Alcaligenes lipid A.
Mice were nasally immunized with PspA with or without Alcaligenes lipid A or cholera toxin three times at one-week intervals. One week after final immunization, lung tissue was prepared, stained with the indicated antibodies and observed by using fluorescence microscopy. Images are representative of two independent experiments. Scale bars: 200 µm.
Supplementary Figure 2

## Slide 3
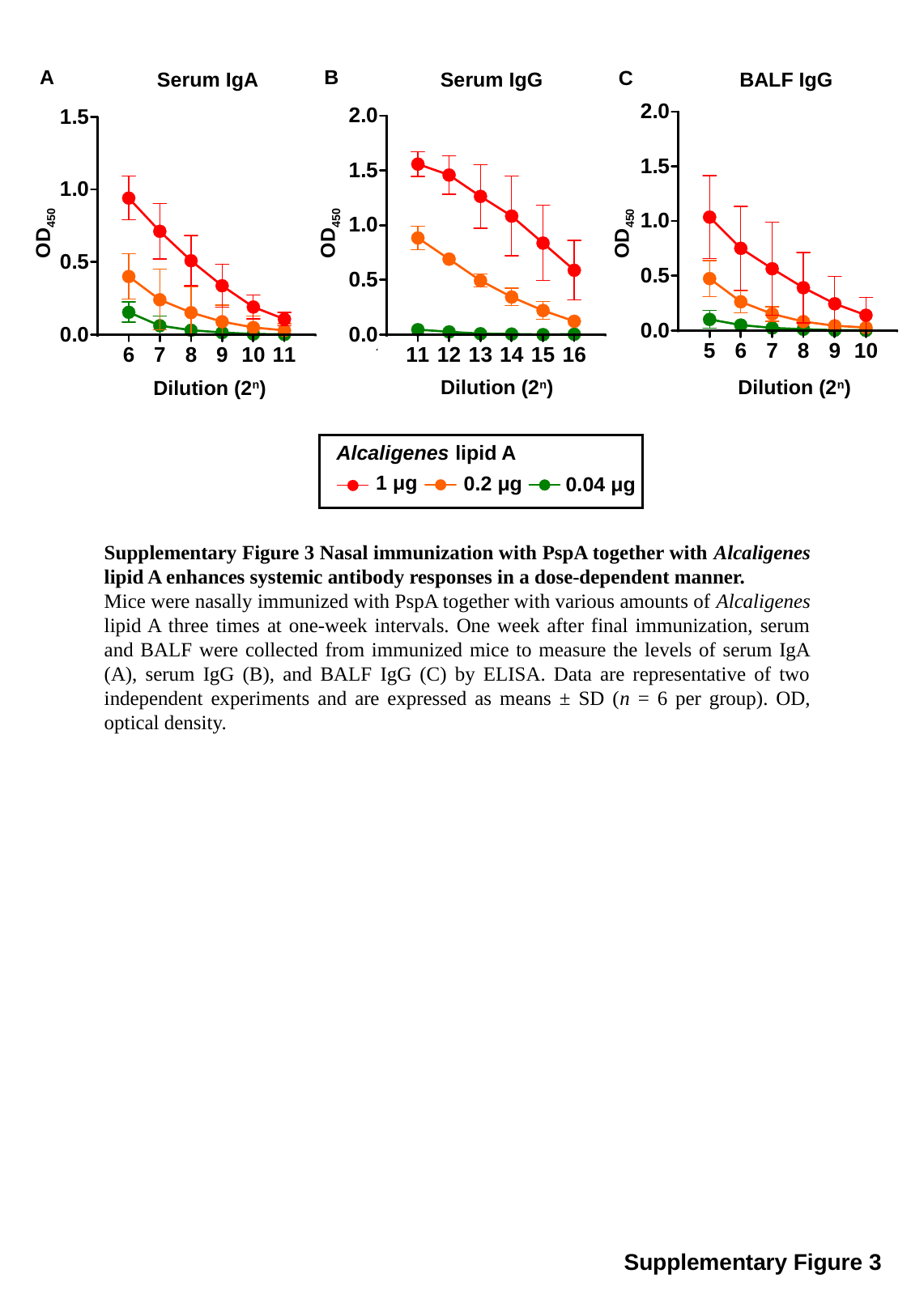

B
A
C
Serum IgA
Serum IgG
BALF IgG
OD450
OD450
OD450
Dilution (2n)
Dilution (2n)
Dilution (2n)
Alcaligenes lipid A
1 μg
0.2 μg
0.04 μg
Supplementary Figure 3 Nasal immunization with PspA together with Alcaligenes lipid A enhances systemic antibody responses in a dose-dependent manner.
Mice were nasally immunized with PspA together with various amounts of Alcaligenes lipid A three times at one-week intervals. One week after final immunization, serum and BALF were collected from immunized mice to measure the levels of serum IgA (A), serum IgG (B), and BALF IgG (C) by ELISA. Data are representative of two independent experiments and are expressed as means ± SD (n = 6 per group). OD, optical density.
Supplementary Figure 3
